# Selective CO_2_ Capture from CO_2_/N_2_ Gas Mixtures Utilizing Tetrabutylammonium Fluoride Hydrates

**DOI:** 10.3390/molecules29061284

**Published:** 2024-03-14

**Authors:** Hyeonjin Kim, Yun-Ho Ahn

**Affiliations:** Department of Chemical Engineering, Soongsil University, 369 Sangdo-ro, Dongjak-gu, Seoul 06978, Republic of Korea

**Keywords:** semi-clathrate hydrates, tetrabutylammonium fluoride, CO_2_/N_2_ separation, selective CO_2_ capture, thermodynamics

## Abstract

Gas hydrates, a type of inclusion compound capable of trapping gas molecules within a lattice structure composed of water molecules, are gaining attention as an environmentally benign gas storage or separation platform. In general, the formation of gas hydrates from water requires high-pressure and low-temperature conditions, resulting in significant energy consumption. In this study, tetrabutylammonium fluoride (TBAF) was utilized as a thermodynamic promoter forming a semi-clathrate-type hydrate, enabling gas capture or separation at room temperature. Those TBAF hydrate systems were explored to check their capability of CO_2_ separation from flue gas, the mixture of CO_2_ and N_2_ gases. The formation rates and gas storage capacities of TBAF hydrates were systematically investigated under various concentrations of CO_2_, and they presented selective CO_2_ capture behavior during the hydrate formation process. The maximum gas storage capacities were achieved at 2.36 and 2.38 mmol/mol for TBAF·29.7 H_2_O and TBAF·32.8 H_2_O hydrate, respectively, after the complete enclathration of the feed gas of CO_2_ (80%) + N_2_ (20%). This study provides sufficient data to support the feasibility of TBAF hydrate systems to be applied to CO_2_ separation from CO_2_/N_2_ gas mixtures based on their CO_2_ selectivity.

## 1. Introduction

The steady increase in greenhouse gas emissions worldwide has highlighted various environmental issues, including climate change [1]. The atmospheric concentration of CO_2_ exceeded 415 ppm in 2022 and is expected to continue rising in the coming years [2]. Consequently, to address the severity of global warming and reduce emissions of carbon dioxide, a 2050 carbon neutrality scenario has been proposed. In order to achieve this goal, research on carbon capture, utilization, and storage (CCUS) is actively underway [3]. Among this, the carbon dioxide capture process incurs the highest cost, necessitating the development of economically viable and efficient capture technology. One of the recently notable technologies is the process of separating mixed gases and extracting desired gases through hydrate formation, known as hydrate-based gas separation (HBGS) [4,5,6].

Clathrate hydrates, crystalline structures formed through water molecule hydrogen bonding, possess the ability to encapsulate low-molecular-weight gas molecules within their lattice framework [7]. They can effectively capture various gases, and their high gas storage capacity per unit volume makes them efficient gas storage media. Particularly notable is their non-explosive nature due to being composed of water. Research has extensively explored the hydrate-based stable storage of energy gases like natural gas [8,9], hydrogen [10,11,12], and their mixtures [13,14,15,16,17], as well as greenhouse gases such as carbon dioxide [18,19,20,21] and nitrous oxide [22,23,24]. In particular, based on the gas capture capability of clathrate hydrates, hydrate-based CO_2_ capture (HBCC), which separates and stores carbon dioxide in hydrate media, has garnered most attention. HBCC offers the advantages of environmental friendliness and high selectivity for carbon dioxide due to its thermodynamically stable characteristics in hydrate [25]. Additionally, HBCC can be designed as a continuous process suitable for large-scale capture and enables cost-effective carbon dioxide treatment. However, traditional hydrate-based technologies typically demand low-temperature, high-pressure conditions, resulting in substantial energy consumption. To address this limitation, this study explores the utilization of ionic species as thermodynamic promoters, enabling hydrate stability even at ambient temperatures.

As previously discussed, pure gas hydrates containing gas species exclusively within the hydrate medium typically necessitate high-pressure and low-temperature conditions to sustain their structural integrity. Consequently, to enhance the feasibility of hydrate-based gas storage techniques, researchers have identified and implemented various thermodynamic promoters. The three most prominent structures of clathrate hydrates are Structure I (sI, comprising two small cages (5^12^) and six large cages (5^12^6^2^)), Structure II (sII, characterized by sixteen small cages (5^12^) and eight large cages (5^12^6^4^)), and Structure H (sH, featuring three small cages (5^12^), two medium cages (4^3^5^6^6^3^), and one large cage (5^12^6^8^)). These thermodynamic promoter molecules typically occupy the large cages of sII (5^12^6^4^) or sH (5^12^6^8^) hydrates, stabilizing the overall structure and enabling secure storage of small gaseous guest molecules within the small cages of sII (5^12^), or the small and medium cages of sH (5^12^, 4^3^5^6^6^3^) hydrates under relatively lower-pressure and higher-temperature conditions. Similarly, certain ionic species can stabilize hydrate structures, leading to the formation of various types of semi-clathrate hydrates. Semi-clathrate hydrates represent unique structures capable of capturing large cations or anions within confined cages, with counterions being incorporated into the hydrate lattice. Typically, the size of the captured ionic species exceeds that of a single large cage of sI (5^12^6^2^) or sII (5^12^6^4^) hydrates, necessitating the partial breaking of cages to accommodate the larger ions. Due to ionic interactions between the captured species in confined cages and the incorporated counterions within the hydrate lattice, some ionic semi-clathrate hydrates exhibit exceptional thermodynamic stability, facilitating the capture of gaseous guest molecules under higher-temperature and lower-pressure conditions [26,27]. This property underscores their potential utility in gas storage and separation applications.

In this study, TBAF hydrates were employed to facilitate the formation of hydrates from mixed gases containing various ratios of carbon dioxide. This study conducted experiments under conditions not previously explored and also confirmed results from a structural change perspective [28,29]. Initially, we investigated the phase equilibrium conditions of TBAF hydrates with different compositions of gas mixtures. Once the temperature and pressure conditions conducive to the formation of TBAF + gas hydrates were confirmed, we conducted an analysis of their structure. Semi-clathrate hydrates exhibit three distinct crystal structures: tetragonal structure-I (TS-I) [10(5^12^)·16(5^12^6^2^)·4(5^12^6^3^)·172 H_2_O], cubic superstructure-I (CSS-I) [2(5^12^)·6(5^12^6^2^)·46 H_2_O], and hexagonal structure-I (HS-I) [3(5^12^)·2(5^12^6^2^)·2(5^12^6^3^)·40 H_2_O] [30]. Since the hydration number determines the crystal structure and chemical properties of ionic hydrates, it was imperative to identify the structures of TBAF·29.7 H_2_O and TBAF·32.8 H_2_O hydrates. Subsequently, we investigated the gas uptake behaviors of TBAF hydrates during the hydrate formation process. The findings of this study yield valuable insights for implementing an energy-efficient carbon dioxide capture and separation process based on semi-clathrate hydrates.

## 2. Results and Discussion

### 2.1. Phase Equilibria of a Semi-Clathrate Hydrate with a Secondary Gaseous Guest

**Figure 1:**
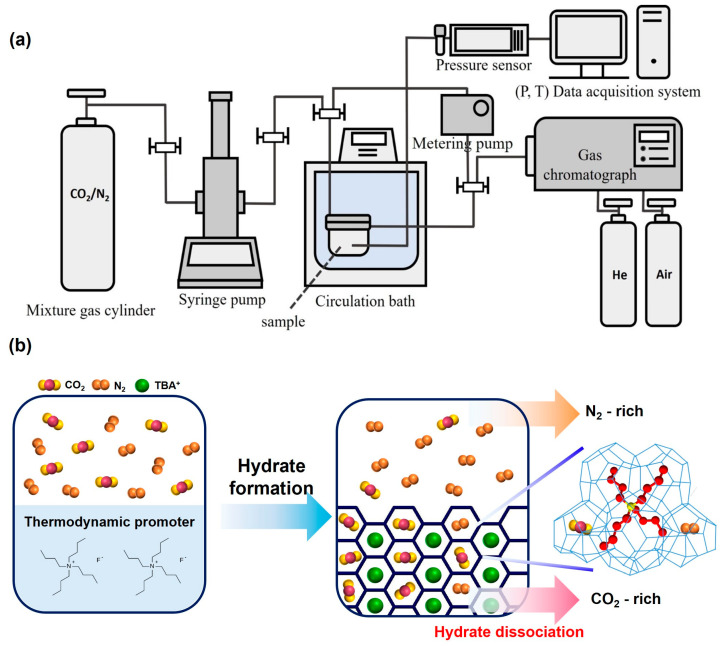
(**a**) Schematic diagram of the overall semi-clathrate hydrate formation system. (**b**) Schematic procedure of TBAF hydrate-based gas separation.

**Figure 2:**
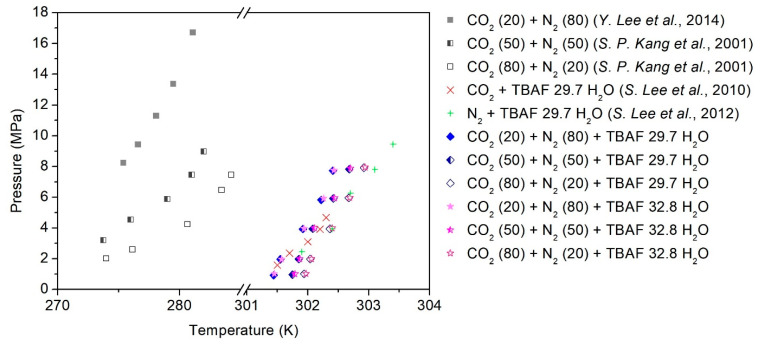
Phase equilibria of the TBAF + CO_2_ (20, 50, 80%) + balanced N_2_ hydrates [31,32,33,34].

The phase equilibria of TBAF-enhanced systems consisting of TBAF·29.7/32.8 H_2_O + CO_2_ (20, 50, 80%) + balanced N_2_ + water were measured using the customized experimental setup (Figure 1), and the detailed procedures are described in Section 4. As depicted in Figure 2, the addition of TBAF had a significant impact on the equilibrium conditions of the gas hydrate system (the detailed temperature and pressure values for each point are summarized in Table 1). Notably, the presence of TBAF caused a notable shift in the equilibrium curve of hydrate formation toward higher temperatures and lower pressures compared to hydrates formed without promoters. This shift implies that under equivalent pressure conditions, hydrate formation becomes achievable at elevated temperatures, and under equivalent temperature conditions, it becomes feasible at reduced pressures. The utilization of TBAF as a thermodynamic promoter resulted in a shift of the equilibrium curve to temperatures, exceeding 30 K higher than that of mixed gas hydrates, thereby enabling the stability of the structure even at ambient conditions. Additionally, akin to hydrates formed without promoters, an increase in the proportion of CO_2_ in the gas mixture led to a rightward shift in the equilibrium curve. This consistent effect across all mixed gases suggests that TBAF exhibited reliable performance. Furthermore, it was observed that there was no significant discrepancy in the equilibrium curve shifts between TBAF·29.7 H_2_O and TBAF·32.8 H_2_O hydrates. Despite the differences in hydration numbers, the thermodynamic promotion effects of TBAF hydrates remained nearly identical, indicating similar crystalline structures and secondary gaseous guest enclathration within the hydrate lattice for both semi-clathrate hydrates.

### 2.2. Spectroscopic Analysis: Structural Identification and Guest Enclathration Behavior

#### 2.2.1. PXRD Analysis of Semi-Clathrate Hydrates

**Figure 3:**
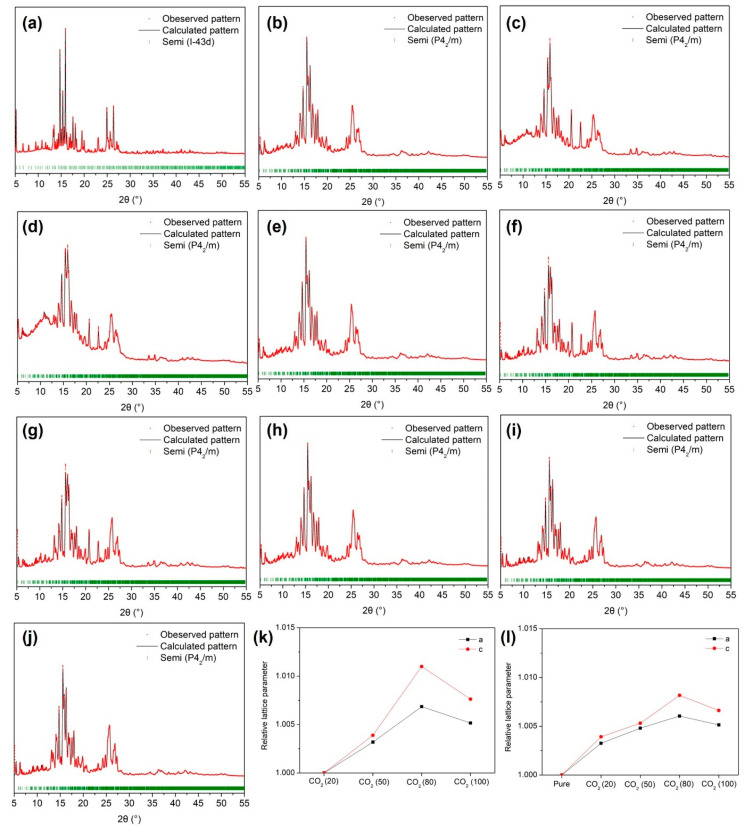
PXRD patterns of the TBAF·29.7 H_2_O hydrates: (**a**) pure; (**b**) CO_2_ (20%) + N_2_ (80%), (**c**) CO_2_ (50%) + N_2_ (50%), (**d**) CO_2_ (80%) + N_2_ (20%), (**e**) CO_2_. PXRD patterns of the TBAF·32.8 H_2_O hydrates: (**f**) pure, (**g**), CO_2_ (20%) + N_2_ (80%), (**h**) CO_2_ (50%) + N_2_ (50%), (**i**) CO_2_ (80%) + N_2_ (20%) (**j**) CO_2_. Relative lattice parameter ratio as a function of CO_2_ composition in the mixed gas: (**k**) TBAF·29.7 H_2_O hydrate, (**l**) TBAF·32.8 H_2_O hydrate.

Understanding the structure of gas hydrates is crucial for accurately predicting and assessing their gas storage capabilities, particularly in applications such as gas separation and storage. In our study, we employed PXRD analysis to delve into the structural characteristics of semi-clathrate hydrates formed under varying compositions of CO_2_ in the feed gas. As depicted in Figure 3a, the TBAF·29.7 H_2_O hydrate devoid of secondary gaseous guests exhibited the anticipated cubic structure of *I-43d*, a well-known configuration. However, upon the introduction of CO_2_ and N_2_ molecules as secondary guests, the semi-clathrate hydrate with an *I-43d* space group transitioned into a tetragonal structure of *P4_2_/m*, as illustrated in Figure 3b–e. In contrast, the tetragonal structure of TBAF·32.8 H_2_O hydrate remained unchanged even under gas pressurization conditions, as depicted in Figure 3f–j. These findings were consistent with the phase equilibria data of TBAF·29.7/32.8 H_2_O + CO_2_ (20, 50, 80%) + balanced N_2_ + water (Figure 2), indicating that despite the differences in hydration numbers, both systems originated from the same crystalline structure. The cubic *I-43d* structure features two small cages (5^12^ cages) per unit cell, suitable for accommodating secondary gaseous guests. In contrast, the tetragonal *P4_2_/m* structure contains ten small cages, offering greater capacity for gaseous guest molecules within the hydrate lattice. Thus, in the presence of CO_2_ and N_2_, the semi-clathrate hydrate of TBAF·29.7 H_2_O favored the formation of the tetragonal *P4_2_/m* structure to accommodate more gaseous guest molecules.

Following the identification of the crystalline structure of TBAF·29.7 H_2_O and TBAF·32.8 H_2_O hydrates, we investigated the lattice parameters obtained from PXRD patterns. Initially, considering the molecular sizes of CO_2_ and N_2_ [35], we anticipated that the lattice parameters would increase proportionally with the CO_2_ composition in the feed gas. However, contrary to expectations, the lattice parameters of both TBAF hydrates increased proportionally with CO_2_ composition up to 80%, but decreased when only CO_2_ was present (i.e., 100% CO_2_ in the feed gas), as shown in Figure 3k,l. To elucidate these results, Raman analysis was conducted.

#### 2.2.2. Raman Analysis of Semi-Clathrate Hydrates

**Figure 4:**
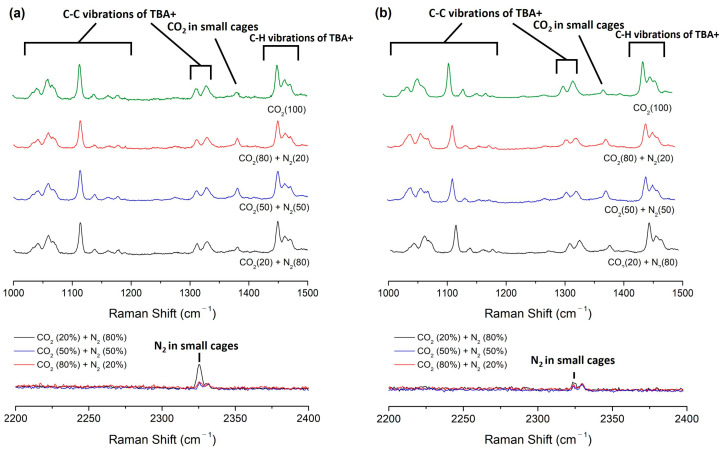
Raman spectra of the TBAF + CO_2_ (20, 50, 80%) + balanced N_2_ hydrates. (**a**) CO_2_ and N_2_ captured in TBAF·29.7 H_2_O hydrates; (**b**) CO_2_ and N_2_ captured in TBAF·32.8 H_2_O hydrates.

Raman spectroscopy was employed to validate the stable enclathration of secondary gaseous guest molecules within the lattice structures of semi-clathrate hydrates [33]. As depicted in Figure 4, confirmation of guest molecule capture in TBAF·29.7/32.8 H_2_O + CO_2_ (20, 50, 80%) + balanced N_2_ hydrate was obtained through comprehensive Raman spectroscopy analysis. In all semi-clathrate hydrates, discernible peaks corresponding to the C−C stretching mode of the tetrabutylammonium cation (TBA^+^) were observed within the range of 950 to 1400 cm^−1^, along with peaks attributed to the C−H bending mode from 1400 to 1500 cm^−1^, indicating the stable formation of TBAF hydrates. Additionally, the presence of CO_2_ and N_2_ within the small cages (5^12^ cages) of semi-clathrate hydrates was evidenced by peaks observed at 1280 and 2324 cm^−1^, respectively.

To further elucidate the PXRD findings described earlier, normalization of Raman peak intensities of CO_2_ and N_2_ was performed using the peak at 1113 cm^−1^, corresponding to the C–C stretching mode of TBA^+^ (Table 2). This normalization process was essential due to the inherent variability in Raman intensity values across measurements. While exact quantitative values from Raman intensity may pose challenges, qualitative comparisons can be made. Notably, as the molar composition of CO_2_ to N_2_ in the feed gas increased, the ratio of Raman peak intensity of enclathrated CO_2_ to N_2_ molecule also showed a corresponding increase in all semi-clathrates. Interestingly, when CO_2_ gas was the sole constituent in the feed gas, the Raman peak intensity of enclathrated CO_2_ appeared almost identical compared to the hydrate formed from a feed gas composition of CO_2_ (80%) + N_2_ (20%). However, in the latter case, additional enclathrated N_2_ was observed within the hydrate lattice, resulting in a larger lattice parameter of the unit cell structure of semi-clathrate hydrates. This finding implies that the presence of N_2_ within the semi-clathrate hydrates contributes to an augmented gas storage capacity compared to scenarios where only CO_2_ is present.

### 2.3. Semi-Clathrate Hydrate-Based Gas Capture and Separation

#### 2.3.1. Gas Uptake during Hydrate Formation

To evaluate the viability of utilizing TBAF hydrates for gas capture and separation processes, we conducted experiments to measure gas storage capacities during hydrate formation from mixed gases. The formation of TBAF hydrates from CO_2_ and N_2_ mixed gas necessitates specific temperature and pressure conditions, which were established based on data obtained from equilibrium experiments (Figure 2). Experimental parameters were set at 40 bar and 299 K with a driving force of ∆T = 3 K. Gas composition inside the reactor was continuously monitored using GC during the hydrate formation experiments, and the number of moles of gases was calculated using the measured temperature and pressure inside the reactor, following the method outlined in Section 3.5. The change in moles of gas in the vapor phase was considered indicative of the moles of gas captured within the hydrate, and thus, the moles of gas captured within the hydrate were determined based on the moles of gas in the vapor phase.

**Figure 5:**
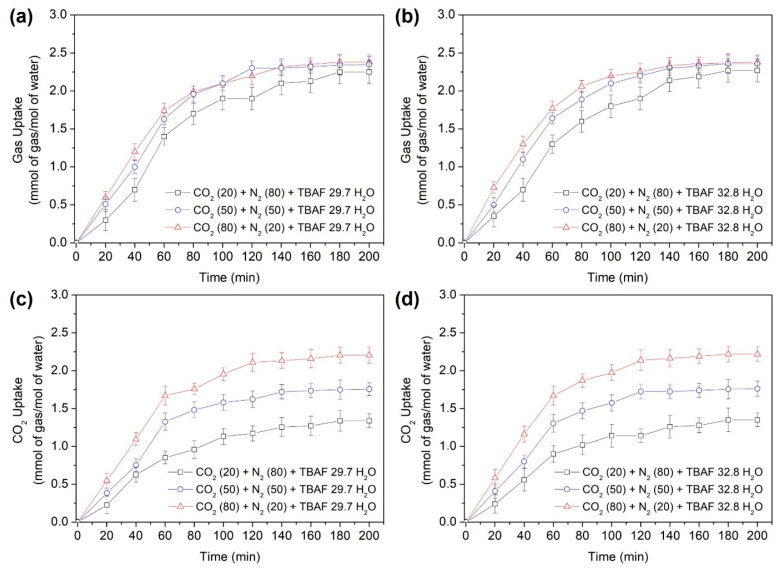
Gas uptake of the semi-clathrate hydrates: (**a**) TBAF·29.7 H_2_O, (**b**) TBAF·32.8 H_2_O; CO_2_ uptake of the semi-clathrate hydrates: (**c**) TBAF·29.7 H_2_O, (**d**) TBAF·32.8 H_2_O.

Figure 5a,b illustrates the overall gas uptake curves of the TBAF + CO_2_ + N_2_ hydrates during hydrate formation over a period of 200 min. The attainment of equilibrium in gas uptake confirms that sufficient hydrate formation occurred within the specified time frame. Gas uptake was expressed as the ratio of consumed gas moles to initially charged water moles. We compared the differences in gas storage capacity based on the composition of mixed gases for the same hydrate, as well as variations in hydrate numbers due to adjustments in mixed gas composition. Following hydrate formation, the total gas storage capacities were measured for each gas mixture: CO_2_ (20%) + N_2_ (80%), CO_2_ (50%) + N_2_ (50%), and CO_2_ (80%) + N_2_ (20%). For the TBAF·29.7 H_2_O hydrate, the gas storage capacities were 2.25, 2.33, and 2.38 mmol/mol, respectively. Similarly, for TBAF·32.8 H_2_O hydrate, the gas storage capacities were 2.25, 2.34, and 2.38 mmol/mol, respectively. Comparing the two TBAF hydrates under the same gas composition revealed similar results. Moreover, this total amount of enclathrated gases corresponded to the tendencies of lattice parameter changes (Figure 3) and the Raman peak intensities of two TBAF hydrates under various mixed gas compositions (Figure 4 and Table 2).

Overall, as the CO_2_ composition in the injected mixed gas increased, the total gas uptake also increased, indicating that CO_2_ is more efficiently captured within the hydrate cages compared to N_2_. This trend is particularly evident from the CO_2_ uptake results illustrated in Figure 5c,d, where the amount of CO_2_ captured within the hydrate increases with a higher CO_2_ composition in the injected gas. Additionally, the storage capacities of the two TBAF hydrates were comparable. When CO_2_ (20%) + N_2_ (80%), CO_2_ (50%) + N_2_ (50%), and CO_2_ (80%) + N_2_ (20%) were injected, the CO_2_ storage after hydrate formation was 1.23, 1.53, and 2.19 mmol/mol, respectively, for the TBAF·29.7 H_2_O hydrate, and 1.23, 1.54, and 2.21 mmol/mol, respectively, for the TBAF·32.8 H_2_O hydrate. This highlights the higher proportion of CO_2_ stored within the hydrate compared to N_2_, resulting in the enhanced total gas storage capacity.

#### 2.3.2. Separation Efficiency of Semi-Clathrate Hydrates-Based Gas Separation

**Figure 6:**
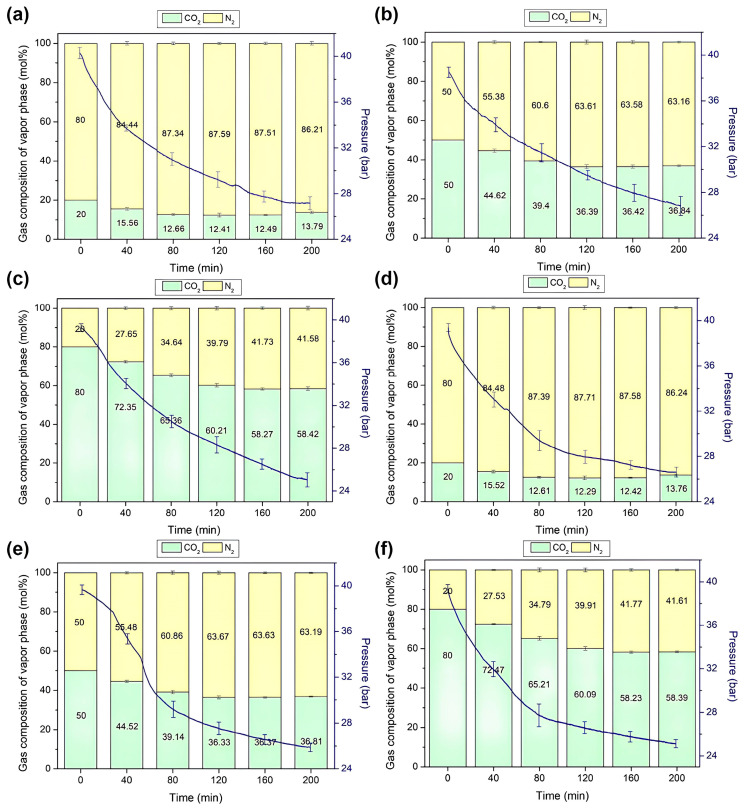
Changes the pressure and gas composition in the vapor phase during the formation of TBAF·29.7 H_2_O hydrates: (**a**) CO_2_ (20%) + N_2_ (80%), (**b**) CO_2_ (50%) + N_2_ (50%), (**c**) CO_2_ (80%) + N_2_ (20%); TBAF·32.8 H_2_O hydrates: (**d**) CO_2_ (20%) + N_2_ (80%), (**e**) CO_2_ (50%) + N_2_ (50%), (**f**) CO_2_ (80%) + N_2_ (20%) over time.

In our quest to comprehend gas separation and storage utilizing semi-clathrate hydrates, we meticulously monitored the changes in gas composition and pressure within the reactor throughout the hydrate formation process. Figure 6 depicts the dynamic evolution of gas composition during hydrate formation from the initial mixed gas for each hydrate, while the gradual decrease in pressure over time attests to the stable capture of gas within the hydrate lattice. This phenomenon underscores the efficacy of gas capture within the hydrate matrix.

Upon pressurizing the TBAF·29.7 H_2_O hydrate with a mixed gas containing 20% CO_2_, an intriguing sequence of events unfolded: initially, CO_2_ was stored within the hydrate lattice, causing a noticeable reduction in CO_2_ composition in the gas phase, as illustrated in Figure 6a. However, around the 160 min mark post-initiation of formation, a discernible uptick in N_2_ uptake appeared, leading to a subsequent increase in CO_2_ composition within the gas phase. Analogous trends were observed for other mixed gases (Figure 6b,c). As the proportion of CO_2_ in the mixed gas increased, the diminishment in CO_2_ concentration within the gas phase became more pronounced, yet over time, N_2_ depletion also became more conspicuous. This intricate interplay elucidates the differential capture kinetics of CO_2_ and N_2_ within the hydrate structure [36,37,38], ultimately impacting separation efficiency.

**Figure 7:**
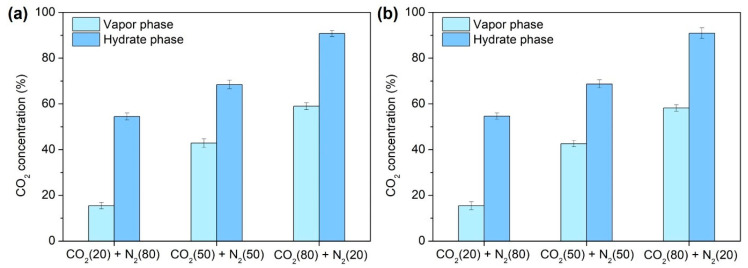
CO_2_ concentration in the vapor phase and hydrate phase after the formation of semi-clathrate hydrates: (**a**) TBAF·29.7 H_2_O, (**b**) TBAF·32.8 H_2_O.

Similarly, TBAF·32.8 H_2_O hydrates exhibited a comparable phenomenon, with CO_2_ preferentially captured initially, followed by N_2_ (Figure 6d–f). Moreover, Figure 7 demonstrates the final CO_2_ concentrations in both the vapor and hydrate phases post-semi-clathrate hydrate formation. Across all compositions, it was revealed that the CO_2_ composition decreased in the vapor phase while concurrently augmenting within the hydrate phase. This disparity underscores the selective separation of CO_2_ from the injected gas via hydrate formation, with a conspicuous augmentation in separation efficiency as the CO_2_ proportion in the mixed gas increased.

Further quantitative analysis yielded two pivotal parameters to gauge the separation efficiency of TBAF hydrates, as tabulated in Table 3. The separation factor presents the extent to which CO_2_ was sequestered relative to other gases in the initial injected gas, while CO_2_ recovery (%) quantifies the proportion of CO_2_ stored from the initial injected gas into the hydrate phase. Encouragingly, the calculation results unveiled highly favorable values for both parameters across various CO_2_ compositions in the mixed gas, with the two TBAF hydrates displaying strikingly similar values. Typically, a separation factor exceeding 10 is deemed substantial [39,40,41,42], underscoring the profound potential application of TBAF hydrates in gas separation and capture technologies, as substantiated by our findings.

## 3. Materials and Methods

### 3.1. Materials

Ultrahigh-purity deionized water was acquired from a Direct-Q3 water purification unit (Millipore, Burlington, MA, USA). The gas mixture of CO_2_ (20, 50, 80%) + balanced N_2_ used for this study was supplied by Seoul Specialty Gas Co. (Seoul, Republic of Korea). TBAF solution with a concentration of 75% in water was purchased from Sigma-Aldrich (St. Louis, MA, USA).

### 3.2. Phase Equilibrium Measurements of a Semi-Clathrate Hydrate

The conventional isochoric method was employed to determine the phase equilibrium conditions of TBAF·29.7 H_2_O and TBAF·32.8 H_2_O with CO_2_ (20, 50, 80%) + balanced N_2_ [43]. The prepared TBAF solutions having a hydration number of TBAF·29.7 H_2_O and TBAF·32.8 H_2_O were loaded into the stirring reactor used for phase equilibrium measurements. The reactor was placed in a circulating water bath (RW-2025G, Jeio Tech., Daejeon, Republic of Korea) capable of temperature control, and mixed gas was pressurized into the reactor to the desired pressure using a syringe pump. The internal temperature of the reactor was maintained at a temperature where hydrate formation does not occur, and air existing inside the cell was flushed out by continuous injection of feed gas for each sample. The stirrer started stirring at 100 rpm. Then, sufficient time was allowed for stabilization without changes in temperature and pressure before starting the temperature changes.

Once the equilibrium state was confirmed without temperature and pressure change, the temperature was gradually lowered to initiate and facilitate the hydrate formation process. After the sudden pressure drop due to the gas enclathration and the completion of the formation process, the temperature was increased very slowly at a rate of 0.1 K/h to provide a sufficient time for an equilibrated dissociation state at each temperature. Pressure and temperature changes during the hydrate formation and dissociation processes were measured using a pressure transducer (PSHB0250BCPG, Sensys Co., Ansan-si, Republic of Korea, full-scale accuracy of ±0.15%, 0–250 bar) and a four-wire type Pt-100 Ω probe (full-scale accuracy of ±0.05%, 13–923 K), respectively, and the data were monitored in real time using a DAQ system (LabVIEW). This data were then utilized to plot the formation-dissociation curve and identify the hydrate equilibrium point at each initial temperature and pressure condition. This procedure was repeated multiple times for each sample to determine the phase equilibrium line of TBAF·29.7 H_2_O and TBAF·32.8 H_2_O with various feed gas compositions.

### 3.3. Structure and Guest Enclathration Analysis of Semi-Clathrate Hydrates

The semi-clathrate hydrate (TBAF + CO_2_ + N_2_) samples formed at 40 bar were prepared for the PXRD and Raman spectroscopic analysis. Prior to retrieving the semi-clathrate hydrate powder samples, the reactor was rapidly cooled using liquid nitrogen to minimize sample damage during the recovery of samples. After a brief period, the reactor was depressurized, and the semi-clathrate hydrates were collected and finely ground to a 40 μm size using a sieve immersed in liquid nitrogen.

Powder X-ray diffraction (PXRD) was utilized to analyze the crystalline structure of semi-clathrate hydrates. The PXRD data were collected at the supramolecular crystallography beamline (2D) facility located at the Pohang Accelerator Laboratory (PAL) in the Republic of Korea. A synchrotron radiation source with a wavelength of 0.900 Å and an ADSC Quantum 210 CCD diffractometer (Pohang, Republic of Korea) were employed for data acquisition. The sample powder, stored in liquid nitrogen, was promptly transferred into pre-cooled polyimide tubing (Cole-Parmer, Vernon Hills, IL, USA) to minimize potential sample damage, and the experiments were conducted at approximately 93 K. The crystal structures of the samples were determined using the Le Bail fitting method, which involves profile matching implemented in the FullProf Program (version 2.00).

Raman spectroscopy was employed to determine the guest enclathration within semi-clathrate hydrates. The Raman spectra of the semi-clathrate hydrates were obtained using a dispersive Raman spectrometer (ARAMIS, Horiba Jobin Yvon Inc., Palaiseau, France), with a 514.53 nm Ar ion laser serving as the excitation source. The scattered light was dispersed by the 1800 grating of the spectrometer and detected by a charge-coupled device (CCD) with electrical cooling (203 K). The sample temperature was maintained at 93 K using a THMSG 600 unit (Linkam Scientific, Redhill, UK).

### 3.4. Gas Composition Analysis

The prepared 100 mL of TBAF solution was injected into the stirring reactor with a volume of 200 mL. Subsequently, the reactor was placed in a circulating water bath. The solution was stirred at a speed of 100 rpm, and mixed gas was pressurized into the reactor to conduct the hydrate formation experiment. The conditions for hydrate formation were set at a pressure of 40 bar, with the formation temperature determined to be 299 K, corresponding to a driving force of ∆T = 3 K at the specified pressure.

To analyze the gas composition inside the hydrate, real-time gas composition in the reactor was measured using a gas chromatograph (GC) (YL6500 GC, Young Lin Instrument Co, Anyang, Republic of Korea) during the hydrate formation process. Gas composition in the reactor was analyzed at 20 min intervals for 200 min, from the pressurization of mixed gas into the reactor to the completion of hydrate formation. To minimize pressure loss due to GC measurements, a metering pump (Eldex, Napa, CA, USA) was used. The constructed hydrate formation system is illustrated in Figure 1.

### 3.5. Quantitative Analysis of Gas Separation Performance

The gas storage capacity within the hydrate was calculated using the gas composition obtained from experiments of semi-clathrate hydrate formation conducted with an in situ GC system. Initially, the critical temperature, critical pressure, and acentric factor of the entire mixed gas were determined by multiplying the factors of each component obtained from the gas composition of the vapor phase analyzed by GC.

(1) $$T_{C}=y_{{CO}_{2}}T_{C,{CO}_{2}}+y_{N_{2}}T_{C,N_{2}}$$

(2) $$P_{C}=y_{{CO}_{2}}P_{C,{CO}_{2}}+y_{N_{2}}P_{C,N_{2}}$$

(3) $$\omega_{C}=y_{{CO}_{2}}\omega_{{CO}_{2}}+y_{N_{2}}\omega_{N_{2}}$$

*T_C_* and *P_C_* are the critical temperature and pressure, respectively, and ω is the acentric factor. These are used to determine the reduced temperature and pressure of the entire mixed gas, as shown below:

(4) $$T_{r}=\frac{T}{T_{C}}$$

(5) $$P_{r}=\frac{P}{P_{C}}$$

$T_{r}\;$and $P_{r}$ present the reduced temperature and pressure, respectively. *T* and *P* present the temperature and pressure obtained through experiment, respectively. We calculated Z by putting the previously obtained parameters into Pitzer’s correlation.

(6) $$B_{0}=0.083-\frac{0.422}{T_{r}^{1.6}}$$

(7) $$B_{1}=0.139-\frac{0.172}{T_{r}^{4.2}}$$

(8) $$Z=Z_{0}+\omega Z_{1}=1+B_{0}\frac{P_{r}}{T_{r}}+{\omega B}_{1}\frac{P_{r}}{T_{r}}$$

$B_{0}$ and $B_{1}$ are the virial coefficients and Z presents the compressibility factor. $Z_{0}$ and $Z_{1}$ are variables related to the correlation for compressibility factors. We used the calculated Z values to determine the total gas storage capacity.

(9) $${\left(∆n\right)}_{t}=n_{0}-n_{t}={\left(\frac{PV}{zRT}\right)}_{0}-{\left(\frac{PV}{zRT}\right)}_{t}$$

${\left(∆n\right)}_{t}$ presents the moles of gas trapped inside the hydrate up to time *t*. *R* is the universal gas constant, and V is the volume of the gas (mL). $n_{t}$, $T_{t}$, $P_{t}$, and $Z_{t}$ present the moles of gas (mol), temperature (K), pressure (bar), and compressibility factor at time *t*, respectively. $n_{0}$, $T_{0}$, $P_{0}$, and $Z_{0}$ present the initial moles of gas (mol), temperature (K), pressure (bar), and compressibility factor of the feed gas, respectively. We used the obtained moles of gas from the above calculation to determine the concentration of CO_2_ in the gas phase and in the hydrate phase after hydrate formation.

(10) $$x_{{CO}_{2}}^{v}=y_{{CO}_{2}}^{1}\times 100(\%)$$

(11) $$x_{{CO}_{2}}^{H}=\frac{n_{gas}^{0}y_{{CO}_{2}}^{0}\times n_{gas}^{1}y_{{CO}_{2}}^{1}}{n_{gas}^{0}\times n_{gas}^{1}}$$

$x_{{CO}_{2}}^{v}$ and $x_{{CO}_{2}}^{H}$ represent the concentration of CO_2_ in the vapor phase and in the hydrate phase, respectively. $n_{gas}^{0}$ is the initial moles of the mixed gas, $n_{gas}^{1}$ is the moles of the mixed gas after hydrate formation, $y_{{CO}_{2}}^{0}$ is the initial moles of CO_2_, and $y_{{CO}_{2}}^{1}$ is the moles of CO_2_ after hydrate formation. Additionally, two parameters were calculated to evaluate the efficiency of gas separation in the mixed gas.

(12) $$S=\frac{n_{{CO}_{2}}^{H}\times n_{N_{2}}^{gas}}{n_{{CO}_{2}}^{gas}\times n_{N_{2}}^{H}}$$

(13) $$R=\frac{n_{{CO}_{2}}^{H}}{n_{{CO}_{2}}^{feed}}\times 100(\%)$$

where S is separation factor, and R is CO_2_ recovery %. $n_{{CO}_{2}}^{H}$ and $n_{N_{2}}^{H}$ present the moles of CO_2_ and N_2_ in the hydrate phase, respectively, $n_{{CO}_{2}}^{gas}$ and $n_{N_{2}}^{gas}$ present the moles of CO_2_ and N_2_ in the vapor phase, respectively, and $n_{{CO}_{2}}^{feed}$ presents the moles of CO_2_ in the feed gas.

## 4. Conclusions

This study investigated the potential of semi-clathrate hydrates containing tetrabutylammonium fluoride (TBAF) for CO_2_/N_2_ gas capture and separation. Structural analysis conducted through PXRD unveiled structural transitions in TBAF·29.7 H_2_O hydrates upon gas capture, suggesting a shift toward structures with a higher number of small cages to accommodate secondary gaseous guest molecules. Raman spectroscopy corroborated the stable enclathration of guest molecules within TBAF hydrates. Moreover, the gas uptake capacities of TBAF hydrates during formation from CO_2_ and N_2_ mixed gases were evaluated. Experimental findings revealed varying gas storage capacities contingent upon the ratio of CO_2_ and N_2_ mixtures, with notably superior storage capacity observed for CO_2_. These results underscore the potential efficacy of TBAF hydrates in the gas separation and capture of CO_2_ and N_2_ mixed gases. Additionally, the separation efficiency of TBAF hydrates was examined, with the observed similarity between the two types of TBAF hydrates underscoring their consistent performance. Hence, this research underscores the significance of TBAF as a promising additive in advancing the practical utilization of semi-clathrate hydrate for gas separation and storage technologies.

## Figures and Tables

**Table 1:** Phase equilibrium data for the TBAF + CO_2_ + N_2_ systems.

Semi-Clathrate Hydrate	Feed Gas					
	CO_2_ (20%) + N_2_ (80%)		CO_2_ (50%) + N_2_ (50%)		CO_2_ (80%) + N_2_ (20%)	
	T/K	P/MPa	T/K	P/MPa	T/K	P/MPa
TBAF·29.7 H_2_O	302.41	7.72	302.68	7.82	302.92	7.92
	302.22	5.82	302.42	5.92	302.67	5.95
	301.92	3.93	302.08	3.95	302.36	3.94
	301.55	1.96	301.85	1.97	302.04	1.98
	301.44	0.92	301.75	0.96	301.94	1.01
TBAF·32.8 H_2_O	302.43	7.75	302.70	7.89	302.94	7.95
	302.26	5.92	302.44	5.95	302.69	5.97
	301.94	3.96	302.11	3.99	302.40	3.99
	301.57	1.96	301.87	1.99	302.06	1.99
	301.45	1.02	301.79	1.01	301.97	1.02

**Table 2:** Raman peak intensity of the guest molecules in semi-clathrate hydrates.

Feed Gas	TBAF·29.7 H_2_O (CO_2_/N_2_)	TBAF·32.8 H_2_O (CO_2_/N_2_)
CO_2_ (20%) + N_2_ (80%)	124.22/63.28	94.79/43.91
CO_2_ (50%) + N_2_ (50%)	163.07/39.13	148.62/36.71
CO_2_ (80%) + N_2_ (20%)	179.69/33.63	158.55/27.42
CO_2_ (100%)	177.16/-	159.20/-

**Table 3:** Separation performance of the semi-clathrate hydrates.

Semi-Clathrate Hydrate	Feed Gas	Separation Factor	CO_2_ Recovery (%)
TBAF·29.7 H_2_O	CO_2_ (20%) + N_2_ (80%)	11.3	62
	CO_2_ (50%) + N_2_ (50%)	15.6	72
	CO_2_ (80%) + N_2_ (20%)	21.1	87
TBAF·32.8 H_2_O	CO_2_ (20%) + N_2_ (80%)	11.4	63
	CO_2_ (50%) + N_2_ (50%)	15.6	73
	CO_2_ (80%) + N_2_ (20%)	21.2	88

## Data Availability

The data presented in this study are openly available.
